# Comparison of central venous minus arterial carbon dioxide pressure to arterial minus central venous oxygen content ratio and lactate levels as predictors of mortality in critically ill patients: a systematic review and meta-analysis

**DOI:** 10.5935/0103-507X.20220026-en

**Published:** 2022

**Authors:** Arnaldo Dubin, Cecilia Inés Loudet, Francisco Javier Hurtado, Mario Omar Pozo, Daniel Comande, Luz Gibbons, Federico Rodriguez Cairoli, Ariel Bardach

**Affiliations:** 1 Intensive Care Service, Sanatorio Otamendi - Buenos Aires, Argentina.; 2 Intensive Care Service, Hospital Interzonal de Agudos General San Martín - La Plata, Argentina.; 3 Intensive Care Service, Hospital Español - ASSE - Montevideo, Uruguay.; 4 Intensive Care Service, Hospital Británico - Buenos Aires, Argentina.; 5 Centro de Investigación de Epidemiología y Salud Pública - Buenos Aires, Argentina.; 6 Instituto de Efectividad Clínica y Sanitaria - Buenos Aires, Argentina.

**Keywords:** Carbon dioxide, Oxygen, Anaerobic metabolism, Lactate, Septic shock, Mortality

## Abstract

**Objective:**

The central venousarterial carbon dioxide pressure to arterial-central venous oxygen content ratio (P_cv-a_CO_2_/C_a-cv_O_2_) is frequently used as a surrogate for tissue oxygenation. We aimed to identify and synthesize literature and quality of evidence supporting P_cv-a_CO_2_/C_a-cv_O_2_ as a predictor of mortality in critically ill patients compared with lactate.

**Methods:**

We searched several databases for studies measuring P_cv-a_CO_2_/C_a-cv_O_2_ in critically ill patients. Independent investigators performed the article screening and data extraction. A random-effects metaanalysis was performed. Pooled standardized mean differences (SMD) were used to compare the prognostic ability of P_cv-a_CO_2_/C_a-cv_O_2_ and lactate.

**Results:**

We initially retrieved 172 studies; 17 were included for qualitative description, and 10 were included for quantitative synthesis. The mean P_cv-a_CO_2_/C_a-cv_O_2_ was higher in nonsurvivors than in survivors (pooled SMD = 0.75; 95%CI 0.34 - 1.17; I^2^ = 83%), as was the case with lactate levels (pooled SMD = 0.94; 95%CI 0.34 - 1.54; I^2^ = 92%). Both tests were statistically significant predictors of mortality, albeit with overlapping 95%CIs between them.

**Conclusion:**

Moderate-quality evidence showed little or no difference in the ability of P_cv-a_CO_2_/C_a-cv_O_2_, compared with lactate, to predict mortality. Nevertheless, our conclusions are limited by the considerable heterogeneity among the studies.

**PROSPERO registration:** CRD42019130387

## INTRODUCTION

In critically ill patients, tissue hypoxia is a leading mechanism of multiple organ failure and death. Therefore, the detection and correction of anaerobic metabolism are crucial tasks. Unfortunately, gold standards for the assessment of tissue oxygenation are lacking. However, blood lactate level is the main variable for tracking hypoperfusion, and it has many drawbacks.^(1)^

In the physiology of exercise, analysis of expired gases allows identification of anaerobic metabolism. Progressive workloads are associated with parallel increases in carbon dioxide production (VCO_2_) and oxygen consumption (VO_2_). The slope of this relationship is the respiratory quotient (RQ = VCO_2_/VO_2_).

Under aerobic conditions, RQ remains unchanged. At some point, however, the increases in VCO_2_ exceed those of VO_2_, and then RQ increases. This inflection point corresponds to the development of hyperlactatemia and is known as the anaerobic threshold.^(2)^ In the other extreme of physiology, during the oxygen supply dependency, the reductions in VO_2_ are higher than the decreases in VCO_2_. Consequently, sharp elevations in RQ ensue.^(3,4)^ In both situations, anaerobic exercise, and critical decreases in O_2_ transport, the underlying phenomenon is the appearance of anaerobic VCO_2_ secondary to bicarbonate buffering of anaerobically generating protons. Thus, an increase in RQ reflects ongoing anaerobic metabolism.

Although the measurement of RQ is an appealing approach, metabolic carts are not usually available in the intensive care unit (ICU) setting. Therefore, some researchers proposed a simplification of Fick’s equation adapted to carbon dioxide (CO_2_), the central venous minus arterial partial pressure of carbon dioxide (PCO_2_) difference to arterial minus central venous O_2_ content ratio (P_cv-a_CO_2_/C_a-cv_O_2_), as a substitute for RQ. Some small observational studies found that a P_cv-a_CO_2_/C_a-cv_O_2_ higher than 1.4 was associated with hyperlactatemia,^(5)^ the presence of oxygen supply dependency,^(6,7)^ and worse outcomes.^(5)^ Moreover, P_cv-a_CO_2_/C_a-cv_O_2_ has been incorporated into some algorithms for tissue oxygenation evaluation and resuscitation.^(8,9)^

The use of P_cv-a_CO_2_/C_a-cv_O_2_ as a surrogate for RQ and tissue oxygenation relies on several assumptions. Some of them are controversial, such as the use of CO_2_ pressure instead of content. Carbon dioxide pressure and content can differ from each other due to changes in the dissociation of CO_2_ from hemoglobin induced by metabolic acidosis, anemia, and the Haldane effect. The Haldane effect is the decrease in CO_2_ binding capacity of hemoglobin with the rise in oxygen saturation. The improvement in blood flow reduces CO_2_ venous content. The associated increase in venous oxygen saturation, however, could blunt the expected decrease in venous PCO_2_ because of a higher dissociation from hemoglobin.^(10,11)^ Another limitation is that central and mixed venous samples are not interchangeable.^(12)^ Furthermore, identification of anaerobic metabolism based on a P_cv-a_CO_2_/C_a-cv_O_2_ cutoff of 1.4 is arbitrary. Since the beginning of anaerobic metabolism is indicated by sharp increases in RQ, not by a particular threshold,^(3,4,10,11,13)^ similar criteria should be considered for P_cv-a_CO_2_/C_a-cv_O_2_. This is especially relevant considering that the normal RQ ranges from 0.67 to 1.30.^(14)^ Moreover, a good correlation between P_cv-a_CO_2_/C_a-cv_O_2_ and RQ has never been shown.

Indeed, experimental studies showed discordant behaviors of P_cv-a_CO_2_/C_a-cv_O_2_ and RQ.^(10,11)^ These studies also showed that P_cv-a_CO_2_/C_a-cv_O_2_ is mainly determined by factors that modify the dissociation of CO_2_ from hemoglobin, such as anemia and metabolic acidosis, not by the RQ. Although P_cv-a_CO_2_/C_a-cv_O_2_ could be a poor surrogate for RQ, it might still be a good predictor of outcome because of the combined effect of anemia and metabolic acidosis on this ratio.

Unlike the aforementioned reports,^(5,7)^ other clinical studies showed that P_cv-a_CO_2_/C_a-cv_O_2_ is a misleading predictor of oxygen supply dependency^(15,16)^ and mortality^(12,17)^ and a useless goal of resuscitation.^(18)^

Despite all these controversies, a systematic review and meta-analysis about the prognostic ability of P_cv-a_CO_2_/C_a-cv_O_2_ is lacking. For this purpose, we analyzed the literature and quality of evidence about this issue. Our research question was whether P_cv-a_CO_2_/C_a-cv_O_2_, compared with lactate levels, is a better predictor of mortality in critically ill patients.

## METHODS

We performed a systematic review and meta-analysis following Cochrane methods and the PRISMA statements for reporting. The review protocol was registered in PROSPERO (CRD42019130387).^(19)^

We included studies performed in critically ill patients who evaluated the prognostic ability of P_cv-a_CO_2_/C_a-cv_O_2_ compared to lactate. The studies comprised the following epidemiological designs: a) cross-sectional studies (calculating odds ratio as measure of effect size and its confidence interval); b) retrospective and prospective cohort studies; c) randomized controlled trials; d) quasiexperimental controlled trials; e) controlled before and after studies; f) uncontrolled before and after studies; g) interrupted time series studies; and h) controlled interrupted time series studies.

We also included studies comparing P_cv-a_CO_2_/C_a-cv_O_2_directed resuscitation with therapies aimed at other goals, such as central venous O_2_ saturation.

We included studies of adults aged ≥ 16 years with different types of shock, postoperative cardiac surgery patients, or other subgroups of critically ill patients in whom P_cv-a_CO_2_/C_a-cv_O_2_ was measured.

The literature search included the following databases: PubMed®, Embase, Cochrane Central Register of Controlled Trials (CENTRAL), *Literatura Latino-Americana e do Caribe em Ciências da Saúde* (LILACS), Cumulative Index to Nursing and Allied Health Literature (CINAHL), and ClinicalTrials.gov. No language restrictions were imposed.

A specialized documentarist librarian conducted the searches. Appendix 1S (Supplementary material) details the search strategies. To avoid missing any study of interest, we also hand-searched the reference lists of primary studies, review articles, and clinical practice guidelines for other important studies. We also inspected the full text of any citation that appeared relevant. Experts on this topic were contacted to check whether there were additional studies to consider in addition to our included studies. The included studies were published in English between 2002 and 2019.

### Data collection and analysis

Citations were uploaded to COVIDENCE, Cochrane’s official web-based platform tool designed to conduct the initial phases of systematic reviews.^(20)^ Duplicate studies were removed. Titles and abstracts of all citations imported were screened independently by three reviewers, and those that were potentially eligible were selected for full text reading. As a second screening phase, the same three reviewers independently analyzed the full texts to confirm that the eligibility criteria were met. Data were abstracted in a prepiloted MS Excel online spreadsheet. We extracted the following information: demographic and clinical data, such as age, sex, comorbidities, type of admission, severity scores at admission (Acute Physiology and Chronic Health Evaluation II - APACHE II, Simplified Acute Physiology Score - SAPS, and Sequential Organ Failure Assessment - SOFA), and laboratory variables; type of study design, methods of selection and inclusion of participants, types of prognostic comparators, and any medical treatments used; mean and standard deviation (SD) of P_cv-a_CO_2_/C_a-cv_O_2_ and lactate. If the studies only reported the median of these variables, the mean and SD were estimated with two simple formulas.^(21)^ For both variables, the first available measurement after ICU admission was considered for the analysis. Other prognostic comparators, language and year of publication and region of origin of the study were also verified.

### Assessment of methodological quality

**Overall risk of bias:** the risk of bias (quality) was assessed by the National Institutes of Health (NIH) Quality Assessment Tool for Observational Cohorts and Cross-Sectional Studies in all studies.^(22)^ Two reviewers independently assessed the risk of bias for each study and resolved any disagreement through discussion and consensus.

The reviewers made an explicit judgment about whether studies had a quality rating of good, fair, or poor, according to the fourteen criteria of the assessment tool (Appendix 2S - Supplementary material).^(22)^

**Assessment of reporting biases:** the search strategy included consultation with leaders in the field, conference and congress proceedings and snowballing techniques to maximize the possibility of finding unpublished studies. We performed funnel plot analyses when eight or more studies were included in each outcome analysis.

**Assessment of heterogeneity and quantitative synthesis**: heterogeneity between studies for each outcome was assessed using Higgins’ I-squared statistic (I^2^). Egger’s test was used to assess asymmetry of the funnel plot to inspect publication bias in continuous data, while Harbord’s test was used for dichotomous outcomes. The results were considered heterogeneous if the I^2^ was > 30%. Forest plots were created to visually show the effect sizes and 95% confidence intervals (95%CI) of outcomes for each study included in the meta-analysis and to show the overall pooled effect with random-effect models. Finally, a sensitivity analysis was performed excluding studies that used mixed venous instead of central venous samples.

Statistical significance was set at p < 0.05, and all tests were two-sided.

Cochrane Collaboration’s statistical software RevMan 5.3^(23)^ was used to perform quantitative synthesis. We provided mean differences (MD) for continuous outcomes measured in the same scales and standardized MD (SMD) for the same outcomes measured by different tests. Due to the impossibility of accessing individual patient data for more precise pooled diagnostic performance estimations and the possible variation in ranges of different biochemical parameters, we used SMDs to compare the prognostic ability of the tests.^(24)^

## RESULTS

**Figure 1:**
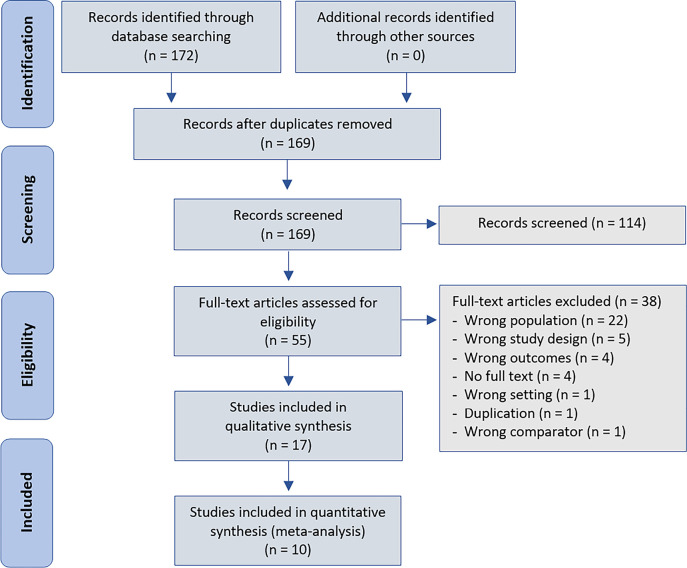
Study selection.

We initially retrieved 172 studies. Seventeen of them, enrolling 1,472 critically ill patients, were included for qualitative (descriptive) synthesis^(5-7,12,15-17,25-34)^, and ten, including 704 patients, were included for quantitative synthesis^(5,12,17,25,27,28,31-34)^ (Figure 1). Out of the final seventeen articles included, four were retrospective observational studies,(5,26,33,34) and thirteen(6,7,12,15-17,25,27-32) were prospective observational studies. The studies included patients with sepsis or septic shock (n = 12),^(6,7,12,17,25-28,31-34)^ cardiovascular surgery (n = 4),^(15,16,29,30)^ and/or requirements of Swan-Ganz catheter monitoring (n = 1).^(5)^

Eight studies were performed in Europe,^(6,7,15,16,28,29,32)^ four in Latin America,(12,25,31,34) and five in Asia.(17,26,27,30,33) The follow-up time was 28 - 30 days in nine studies,(5,7,12,17,25,26,28,31,33) until ICU discharge in two,^(32,34)^ until hospital discharge in one,^(29)^ and unspecified in five of them.^(6,15,16,27,30)^ Appendix 3S (Supplementary material) shows data of the seventeen included studies for qualitative synthesis, including the type of study, sample size, type of participants, variable of interest, comparator, main outcomes, and main results.

### P_cv-a_CO_2_/C_a-cv_O_2_ and lactate as mortality predictors

Ten articles provided information about values of P_cv-a_CO_2_/C_a-cv_O_2_ and lactate in survivors and nonsurvivors mainly from septic shock and were grouped for performing metanalysis.^(5,12,17,25,27,28,31-34)^ In the quantitative synthesis, lactate levels were significantly higher in nonsurvivors than in survivors (pooled SMD = 95%CI 0.94; 0.34 - 1.54; p < 0.00001). We found considerable statistical heterogeneity (I^2^ = 92%). P_cv-a_CO_2_/C_a-cv_O_2_ was also significantly higher in nonsurvivors (pooled SMD = 95%CI 0.75; 0.34 - 1.17; p < 0.00001). We also found considerable statistical heterogeneity (I^2^ = 83%).

**Figure 2:**
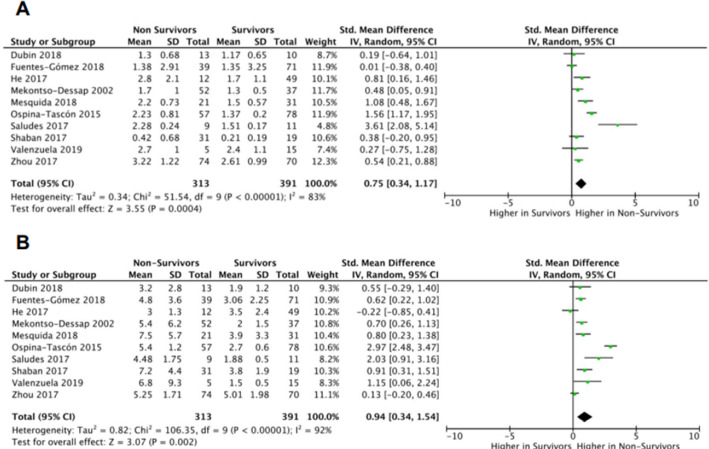
Forest plots of P_cv-a_CO_2_/C_a-cv_O_2_ (A) and lactate (B) in survivors and nonsurvivors.

Figure 2 shows the forest plots of both variables. Sensitivity analysis excluding the studies that used mixed venous instead of central venous samples are shown in appendix 4S (Supplementary material). Forest plots of P_cv-a_CO_2_/C_a-cv_O_2_ and lactate also showed that both levels were significantly higher in nonsurvivors than in survivors (Appendix 5S - Supplementary material).

### Risk of bias in the included studies

We assessed fourteen domains of possible biases, according to prespecified criteria. Details for each included study are provided in table 1. Overall, only five studies were classified with a quality rating of “good”.^(15-17,26,29)^ Within this group, only one study had a low risk of bias in 13 of the 14 domains evaluated.^(29)^ Eleven studies were classified with a quality rating of “fair”,^(5-7,12,25,27,30-34)^ and the remaining studies were classified as “poor”.^(28)^ The domains with more “high risk of bias” were blinding of outcome assessors, sample size justification, statistical analyses (key potential confounding variables measured and adjusted statistically), and study population.

**Table 1:** The risk of bias (quality) was assessed by the NIH Quality Assessment Tool for Observational Cohort and Cross-Sectional Studies^(22)^

Risk of bias-NIH checklist	1	2	3	4	5	6	7	8	9	10	11	12	13	14	Overall quality
Mekontso-Dessap et al.^(5)^	√	±	±	±	Ø	√	√	√	√	±	√	Ø	±	Ø	Fair
Monnet et al.^(6)^	√	Ø	±	±	Ø	√	√	√	√	√	Ø	Ø	√	Ø	Fair
Mallat et al.^(7)^	√	Ø	±	±	√	√	√	√	√	√	Ø	Ø	√	Ø	Fair
Dubin et al.^(12)^	√	Ø	±	±	Ø	√	√	√	√	√	√	Ø	√	√	Fair
Abou-Arab et al.^(15)^	√	√	±	√	√	√	√	√	√	√	Ø	Ø	√	Ø	Good
Fischer et al.^(16)^	√	√	√	√	Ø	√	√	√	√	√	Ø	Ø	√	Ø	Good
Shaban et al.^(17)^	√	√	±	√	Ø	√	√	√	√	√	√	Ø	√	Ø	Good
Valenzuela Espinoza et al.^(25)^	√	Ø	±	±	√	√	√	√	√	√	√	Ø	√	Ø	Fair
Gao et al.^(26)^	√	√	±	√	Ø	√	√	√	√	√	√	Ø	±	±	Good
He et al.^(27)^	√	√	±	√	Ø	√	√	√	Ø	√	√	Ø	√	√	Fair
Mesquida et al.^(28)^	√	Ø	±	±	√	√	√	√	Ø	Ø	√	Ø	±	Ø	Poor
Moussa et al.^(29)^*	√	√	√	√	√	√	√	√	√	√	√	Ø	√	√	Good
Mukai et al.^(30)^*	√	Ø	±	±	√	√	√	√	√	√	√	Ø	√	√	Fair
Ospina-Tascón et al.^(31)^	√	Ø	±	±	Ø	√	√	√	√	√	√	Ø	√	√	Fair
Saludes et al.^(32)^	√	Ø	±	±	Ø	√	√	√	√	√	Ø	Ø	√	Ø	Fair
Zhou et al.^(33)^	√	√	±	√	Ø	√	√	√	Ø	√	√	Ø	√	√	Fair
Fuentes-Gómez et al.^(34)^	√	Ø	±	±	Ø	√	√	√	√	√	√	Ø	±	Ø	Fair

√ - yes; ± - cannot determine, not applicable, or not reported; Ø - no.

### Assessment of reporting bias

**Figure 3:**
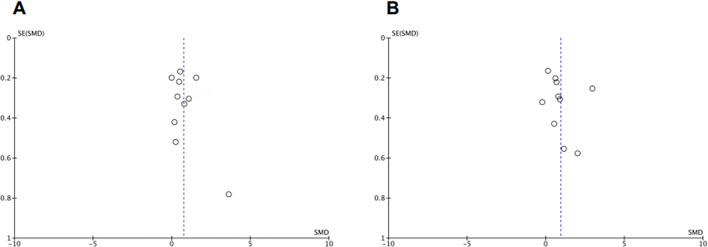
Funnel plots of P_cv-a_CO_2_/C_a-cv_O_2_ (A) and arterial lactate (B) in survivors and nonsurvivors.

The funnel plots show a slight asymmetry that is probably explained by the methodological heterogeneity between studies. There is no strong evidence of publication bias due to the absence of a marked asymmetry in these graphs. Egger’s regression test p values are higher than 0.1, excluding the presence of this type of bias. Funnel plots for P_cv-a_CO_2_/C_a-cv_O_2_ and lactate are shown in figure 3. For P_cv-a_CO_2_/C_a-cv_O_2_, Higgins’ I^2^ (inconsistency) was 83% (95% CI=69% to 89%). The Begg-Mazumdar p value was 0.73, and Egger’s test’s p value was 0.48. For lactate, I^2^ was 92% (95%CI = 87% to 94%). The Begg-Mazumdar p value was 0.11, and Egger’s test’s p value was 0.46.

Funnel plots for P_cv-a_CO_2_/C_a-cv_O_2_ and lactate, excluding studies that used mixed venous samples, are shown in appendix 4S (Supplementary material).

## DISCUSSION

P_cv-a_CO_2_/C_a-cv_O_2_ is frequently used as a surrogate for tissue oxygenation and a predictor of outcome in critically ill patients. In this systematic review and meta-analysis, we aimed to identify the evidence supporting the ability of this variable to predict mortality and to contrast it with the performance of lactate levels. We found that P_cv-a_CO_2_/C_a-cv_O_2_ was positively associated with increased mortality. This prognostic ability, however, was not better than that of lactate.

All included articles confirmed that P_cv-a_CO_2_/C_a-cv_O_2_ is a predictor of mortality. Mesquida et al.^(28)^ reported an area under the receiver operating characteristic (AUROC) of P_cv-a_CO_2_/C_a-cv_O_2_ better than that of lactate levels. In addition, Zhou et al.^(33)^ showed that the AUROC of P_cv-a_CO_2_/C_a-cv_O_2_ combined with lactate was significantly higher than lactate alone. Although P_cv-a_CO_2_/C_a-cv_O_2_ and lactate are positively associated with worst outcomes, currently published data do not allow us to interpret which of these two variables is a better predictor of mortality. Nevertheless, we approximated an estimate by comparing the pooled SMDs of lactate and P_cv-a_CO_2_/C_a-cv_O_2_ to evaluate the strength of their association with ICU mortality. Unlike the MD, which measures the absolute difference between the mean values of two groups, the SMD is the optimal statistic to use when outcome measurements have different scales. The SMD expresses the size of the intervention effect in each study relative to the between-participant variability in the outcome measurements observed in that study.^(24)^ A higher value of the SMD indicates a stronger association with the outcome. In our study, the SMD of lactate was higher than that of P_cv-a_CO_2_/C_a-cv_O_2_. Consequently, the former shows a trend toward a higher association with mortality than the latter. The confidence intervals, however, overlapped. Thus, the wide and overlapping confidence intervals preclude the possibility of drawing definitive conclusions from these observations.

Our study has many strengths, such as the registration of the protocol in PROSPERO, the exhaustive search strategy used, and diversity of epidemiological study designs considered the risk of bias assessment (quality), and we used random-effect models for performing meta-analysis.

As limitations, all studies included in the qualitative and quantitative analysis were observational, and many of them did not report the operative characteristics of markers as diagnostic tests. Unfortunately, some studies did not show enough clinical information. Thus, selection and information bias could be present because of different populations, characteristics of the critically ill patients, variable definitions, and study designs. Accordingly, we found a high degree of heterogeneity (I^2^ = 84%) in the quantitative analysis of the P_cv-a_CO_2_/C_a-cv_O_2_ performance. This heterogeneity was even greater for lactate (I^2^ = 92%). As mentioned, we speculate that this heterogeneity may be explained by differences in the clinical characteristics of the recruited patients and in study designs. The differences in the definition of mortality among the studies - ICU, hospital, Day 28, and Day 30 - might also have contributed to the high heterogeneity. Nevertheless, we speculate that the effect was only minor because all criteria involved shortterm mortality.

Although we analyzed the studies by means of the SMD, we observed important variability in the magnitude of the SDs. Since we were unable to access the original datasets of the included studies to perform an individual patient-level meta-analysis, we had to resort to aggregated data.

The cause of the association between P_cv-a_CO_2_/C_a-cv_O_2_ and patient outcomes is another relevant issue. While P_cv-a_CO_2_/C_a-cv_O_2_ is considered a surrogate for tissue oxygenation, a clinical correlation between this variable and RQ has never been shown. Unfortunately, there are no clinical studies comparing P_cv-a_CO_2_/C_a-cv_O_2_ with RQ. In surgical patients, RQ, but not P_cv-a_CO_2_/C_a-cv_O_2,_ was a predictor of complications. In addition, RQ and P_cv-a_CO_2_/C_a-cv_O_2_ were not correlated.^(35)^ On the other hand, experimental studies showed that P_cv-a_CO_2_/C_a-cv_O_2_ is more dependent on factors modifying the CO_2_ dissociation curve than on RQ.^(10,11)^ Given that P_cv-a_CO_2_/C_a-cv_O_2_ is a composite variable, it could be a predictor of mortality even in the absence of tissue hypoxia and changes in the RQ. Then, the presence of anemia and metabolic acidosis, which are predictors of mortality in critically ill patients, could themselves explain the predictive ability of P_cv-a_CO_2_/C_a-cv_O_2_.^(36,37)^

## CONCLUSION

This systematic review and meta-analysis showed moderate-quality evidence of little or no difference in the ability of P_cv-a_CO_2_/C_a-cv_O_2_ compared with lactate to predict mortality. Nevertheless, our conclusions are limited by the considerable heterogeneity among the studies.
