# Supplementary material for: Comparison of central venous minus arterial carbon dioxide pressure to arterial minus central venous oxygen content ratio and lactate levels as predictors of mortality in critically ill patients: a systematic review and meta-analysis
Source: Rev Bras Ter Intensiva. 2022 Apr-Jun;34(2):279–86. doi: 10.5935/0103-507X.20220026-en (PMC9354115; doi:10.5935/0103-507X.20220026-en)

Comparison of central venous minus arterial carbon dioxide pressure to arterial minus central venous oxygen content ratio and lactate levels as predictors of mortality in critically ill patients: a systematic review and meta-analysis

Comparação da proporção entre pressão venosa central menos arterial de dióxido de carbono e conteúdo de oxigênio arterial menos venoso central e níveis de lactato como preditores de mortalidade em pacientes críticos: uma revisão sistemática e metanálise

Arnaldo Dubin[1], Cecilia Inés Loudet[2], Francisco Javier Hurtado[3], Mario Omar Pozo[4], Daniel Comande[5], Luz Gibbons[5], Federico Rodriguez Cairoli[5], Ariel Bardach[6]

Appendix 1S - Search strategies

Search	Query	Results
#28	Search (#13 AND #27)	100
#27	Search (#14 OR #15 OR #16 OR #17 OR #18 OR #19 OR #20 OR #21 OR #22 OR #23 OR #24 OR #25 OR #26)	273987
#26	Search Shocked Patient*[tiab]	144
#25	Search Shock Patient*[tiab]	1577
#24	Search Shock Unit*[tiab]	13
#23	Search Critical Care*[tiab]	27007
#22	Search Intensive Care*[tiab]	129590
#21	Search Stressed[tiab]	40610
#20	Search Critically Ill[tiab]	39754
#19	Search Critical Ill*[tiab]	8479
#18	Search Coronary Care*[tiab]	4869
#17	Search ICU[tiab]	48816
#16	Search Intensive Care Units[Mesh]	75831
#15	Search Critical Care[Mesh]	53615
#14	Search Critical Illness[Mesh]	25203
#13	Search (#1 OR #2 OR #3 OR #4 OR #5 OR #6 OR #7 OR #8 OR #9 OR #10 OR #11 OR #12)	617
#12	Search Arterial-PCO2 Tension[tiab]	102
#11	Search Arterial-CO2 Tension[tiab]	280
#10	Search “P(v-a)CO2”[tiab]	17
#9	Search “Pv-aCO2”[tiab]	23
#8	Search Venous-Arterial Carbon[tiab]	47
#7	Search Venous-To-Arterial CO2[tiab]	35
#6	Search Venoarterial CO2[tiab]	57
#5	Search Venoarterial Carbon[tiab]	55
#4	Search Venous-Arterial CO2[tiab]	34
#3	Search Venous-Arterial PCO2[tiab]	24
#2	Search Venous-To-Arterial PCO2[tiab]	23
#1	Search Venoarterial PCO2[tiab]	37

EMBase (Elsevier) 26-02-2019

No.	Query	Results
#28	#13 AND #27	65
#27	#14 OR #15 OR #16 OR #17 OR #18 OR #19 OR #20 OR #21 OR #22 OR #23 OR #24 OR #25 OR #26	896687
#26	'shocked patient':ti,ab OR 'shocked patients':ti,ab	219
#25	'shock patient':ti,ab OR 'shock patients':ti,ab	3252
#24	'shock unit':ti,ab OR 'shock units':ti,ab	16
#23	'critical care':ti,ab OR 'critical cares':ti,ab	41211
#22	'intensive care':ti,ab OR 'intensive cares':ti,ab	184496
#21	stressed:ti,ab	51437
#20	'critically ill':ti,ab	58355
#19	'critical ill':ti,ab OR 'critical illness':ti,ab	11432
#18	'coronary care':ti,ab	6807
#17	icu:ti,ab	98816
#16	'intensive care unit'/exp	164841
#15	'intensive care'/exp	651904
#14	'critical illness'/exp	26874
#13	#1 OR #2 OR #3 OR #4 OR #5 OR #6 OR #7 OR #8 OR #9 OR #10 OR #11 OR #12	146
#12	'arterial pco2 tension':ti,ab	0
#11	'arterial co2 tension':ti,ab	49
#10	'p(v-a)co2':ti,ab	7
#9	'pv-aco2':ti,ab	15
#8	'venous arterial carbon':ti,ab	31
#7	'venous to arterial co2':ti,ab	5
#6	'venoarterial co2':ti,ab	4
#5	'venoarterial carbon':ti,ab	18
#4	'venous arterial co2':ti,ab	9
#3	'venous arterial pco2':ti,ab	14
#2	'venous to arterial pco2':ti,ab	5
#1	'venoarterial pco2':ti,ab	2

Cochrane Library (Wiley) 7-03-2019

ID	Search	Hits
#1	Venoarterial PCO2:ti,ab,kw	5
#2	Venous-To-Arterial PCO2:ti,ab,kw	0
#3	Venous-Arterial PCO2:ti,ab,kw	5
#4	Venous-Arterial CO2:ti,ab,kw	6
#5	Venoarterial Carbon:ti,ab,kw	7
#6	Venoarterial CO2:ti,ab,kw	7
#7	Venous-To-Arterial CO2:ti,ab,kw	2
#8	Venous-Arterial Carbon:ti,ab,kw	8
#9	Pv-aCO2:ti,ab,kw	0
#10	"P(v-a)CO2":ti,ab,kw	1
#11	Arterial-CO2 Tension:ti,ab,kw	42
#12	"Arterial-PCO2 Tension":ti,ab,kw	0
#13	#1 OR #2 OR #3 OR #4 OR #5 OR #6 OR #7 OR #8 OR #9 OR #10 OR #11 OR #12	66
#14	MeSH descriptor: [Critical Illness] explode all trees	1829
#15	MeSH descriptor: [Critical Care] explode all trees	1911
#16	MeSH descriptor: [Intensive Care Units] explode all trees	3233
#17	ICU:ti,ab,kw	6897
#18	Coronary Care*:ti,ab,kw	6945
#19	Critical Ill*:ti,ab,kw	5856
#20	Critically Ill:ti,ab,kw	5055
#21	Stressed:ti,ab,kw	762
#22	Intensive Care*:ti,ab,kw	22693
#23	Shock Unit*:ti,ab,kw	1429
#24	Shock Patient*:ti,ab,kw	6282
#25	Shocked Patient*:ti,ab,kw	33
#26	Critical Care*:ti,ab,kw	11779
#27	#14 OR #15 OR #16 OR #17 OR #18 OR #19 OR #20 OR #21 OR #22 OR #23 OR #24 OR #25 OR #26	43428
#28	#13 AND #27	20

CINAHL (EBSCO) 7-03-2019

#	Query	Results
S25	S11 AND S24	24
S24	S12 OR S13 OR S14 OR S15 OR S16 OR S17 OR S18 OR S19 OR S20 OR S21 OR S22 OR S23	95,254
S23	TI Shocked Patient* OR AB Shocked Patient*	138
S22	TI Shock Patient* OR AB Shock Patient*	11,200
S21	TI Shock Unit* OR AB Shock Unit*	2,380
S20	TI Intensive Care* OR AB Intensive Care*	55,897
S19	TI Stressed OR AB Stressed	4,018
S18	TI Critically Ill OR AB Critically Ill	17,441
S17	TI Critical Ill* OR AB Critical Ill*	12,405
S16	TI Coronary Care* OR AB Coronary Care*	8,036
S15	TI ICU OR AB ICU	23,034
S14	(MH "Intensive Care Units+")	51,328
S13	(MH "Critical Care+")	24,537
S12	(MM "Critical Illness")	6,372
S11	S1 OR S2 OR S3 OR S4 OR S5 OR S6 OR S7 OR S8 OR S9 OR S10	51
S10	TI Arterial-PCO2 Tension OR AB Arterial-PCO2 Tension	0
S9	TI Arterial-CO2 Tension OR AB Arterial-CO2 Tension	0
S8	TI "P(v-a)CO2" OR AB "P(v-a)CO2"	8
S7	TI "Pv-aCO2" OR AB "Pv-aCO2"	6
S6	TI Venous-Arterial Carbon OR AB Venous-Arterial Carbon	11
S5	TI Venoarterial CO2 OR AB Venoarterial CO2	6
S4	TI Venoarterial Carbon OR AB Venoarterial Carbon	10
S3	TI Venous-Arterial CO2 OR AB Venous-Arterial CO2	7
S2	TI Venous-To-Arterial PCO2 OR AB Venous-To-Arterial PCO2	14
S1	TI Venoarterial PCO2 OR AB Venoarterial PCO2	1

ClinicalTrials.gov 7-03-2019

7 Studies found for: Venoarterial PCO_2 OR Venous-To-Arterial PCO_2 OR Venous-Arterial PCO_2 OR Venous-Arterial CO_2 OR Venoarterial Carbon OR Venoarterial CO_2 OR Venous-To-Arterial CO2 | Critically Ill OR Stressed OR Critical Illness OR Shock

Appendix 2S - Assessment of the risk of bias (quality) by the NIH Quality Assessment Tool for Observational Cohort and Cross-Sectional Studies.

Study Quality Assessment Tools (NIH Study Quality Assessment Tools. https://www.nhlbi.nih.gov/health-topics/study-quality-assessment-tools)

Criteria

1. Was the research question or objective in this paper clearly stated?
2. Was the study population clearly specified and defined?
3. Was the participation rate of eligible persons at least 50%?
4. Were all the subjects selected or recruited from the same or similar populations (including the same time period)? Were inclusion and exclusion criteria for being in the study prespecified and applied uniformly to all participants?
5. Was a sample size justification, power description, or variance and effect estimates provided?
6. For the analyses in this paper, were the exposure(s) of interest measured prior to the outcome(s) being measured?
7. Was the timeframe sufficient so that one could reasonably expect to see an association between exposure and outcome if it existed?
8. For exposures that can vary in amount or level, did the study examine different levels of the exposure as related to the outcome (e.g., categories of exposure, or exposure measured as continuous variable)?
9. Were the exposure measures (independent variables) clearly defined, valid, reliable, and implemented consistently across all study participants?
10. Was the exposure(s) assessed more than once over time?
11. Were the outcome measures (dependent variables) clearly defined, valid, reliable, and implemented consistently across all study participants?
12. Were the outcome assessors blinded to the exposure status of participants?
13. Was loss to follow-up after baseline 20% or less?
14. Were key potential confounding variables measured and adjusted statistically for their impact on the relationship between exposure(s) and outcome(s)?

Appendix 3S - Data of the seventeen included studies for qualitative synthesis, including the type of study, sample size, type of participants, variable of interest, comparator, main outcomes and main results.

Table 1S - Characteristics of studies and results

Reference	Study design	N	Type of ICU-population	Variables of interest	Comparator	Main outcome	Main results
Mekontso-Dessap et al.[5]	Retrospective cohort	89	Patients requiring Swan-Ganz	$P_{mv\text{-}a}CO_2/C_{a\text{-}mv}O_2$	Lactate $C_{a\text{-}mv}O_2$ $S_{mv}O_2$	30-day mortality	$P_{mv\text{-}a}CO_2/C_{a\text{-}mv}O_2$ had its best correlation with lactate ($r = 0.57$, $p < 0.0001$) and the best AUROC for lactate $\geq$ 2.0mmol/L. Survivors had lower lactate than non-survivors (2.0 ± 1.5 *versus* 5.4 ± 6.1mmol/L, $p < 0.01$) but $P_{mv\text{-}a}CO_2/C_{a\text{-}mv}O_2$ was not different (1.3 ± 0.5 *versus* 1.7 ± 1.0, $p = 0.07$). Higher survival for patients with $P_{mv\text{-}a}CO_2/C_{a\text{-}mv}O_2 > 1.4$ than ≤ 1.4 (38 ± 10 *versus* 20 ± 8%, $p < 0.01$)
Monnet et al.[6]	Prospective cohort	25	Shock of any etiology	$P_{cv\text{-}a}CO_2/C_{a\text{-}cv}O_2$	Lactate $S_{cv}O_2$	15% increase in VO_2	$P_{cv\text{-}a}CO_2/C_{a\text{-}cv}O_2$ correlated with lactate ($r = 0.56$, $p < 0.0001$) In the whole group, $S_{cv}O_2$, lactate, $P_{cv\text{-}a}CO_2/C_{a\text{-}cv}O_2$ failed to predict the increase in VO_2 (AUROC not significantly different from 0.5). In volume responders ($n = 25$), $P_{cv\text{-}a}CO_2/C_{a\text{-}cv}O_2$ and lactate, but not $S_{cv}O_2$ were higher in VO_2 responders than in non-responders (2.3 ± 0.8 *versus* 1.3 ± 0.5, 5.5 ± 4.0 *versus* 2.3 ± 1.1mmol/L, and 70 ± 15 *versus* 64 ± 4%). AUROCs were 0.94 ± 0.05, 0.91 ± 0.06, and 0.68 ± 0.11
Mallat et al.[7]	Prospective cohort	51	Septic shock	$P_{cv\text{-}a}CO_2/C_{a\text{-}cv}O_2$	Lactate $S_{cv}O_2$	15% increase in VO_2	Lactate correlated with $P_{cv\text{-}a}CO_2/C_{a\text{-}cv}O_2$ ($r = 0.33$, $p = 0.001$). In volume responders ($n = 51$), AUROCs of $S_{cv}O_2$, lactate, $P_{cv\text{-}a}CO_2/C_{a\text{-}cv}O_2$ for VO_2 were 0.624, 0.745, and 0.962.
Dubin et al.[12]	Prospective cohort	23	Septic shock	$P_{cv\text{-}a}CO_2/C_{a\text{-}cv}O_2$	Lactate	ICU and hospital mortality	Similar $P_{cv\text{-}a}CO_2/C_{a\text{-}cv}O_2$ (1.17 ± 0.65 *versus* 1.30 ± 0.68, $p = 0.65$) and lactate (1.9 ± 1.2 versus 3.2 ± 2.8mmol/L, $p = 0.16$) in survivors and non-survivors. $P_{cv\text{-}a}CO_2/C_{a\text{-}cv}O_2$ and lactate correlated ($r^2 = 0.38$, $p < 0.01$)
Abou-Arab et al.[15]	Prospective cohort	92	Cardiac surgery	$P_{cv\text{-}a}CO_2/C_{a\text{-}cv}O_2$	Lactate $S_{cv}O_2$	15% increase in VO_2	Lactate did not correlate with $P_{cv\text{-}a}CO_2/C_{a\text{-}cv}O_2$ ($r = 0.05$, $p = 0.59$). $P_{cv\text{-}a}CO_2/C_{a\text{-}cv}O_2$ and lactate were similar in responders and non-responders (1.93 [1.36 - 2.29] *versus* 1.89 [1.42 - 2.0], $p = 0.71$ and 1.8 ± 0.9 *versus* 1.9 ± 0.7mmol/L, $p = 0.59$). $S_{cv}O_2$ was higher in responders (68 ± 12 *versus* 61 ± 10%, $p = 0.003$). AUROC of $P_{cv\text{-}a}CO_2/C_{a\text{-}cv}O_2$ and $S_{cv}O_2$ were 0.53 [0.4 - 0.65], $p = 0.71$ and 0.67 [0.55 - 0.78], $p < 0.0001$.
Fischer et al.[16]	Prospective cohort	17	Cardiac surgery	$P_{cv\text{-}a}CO_2/C_{a\text{-}cv}O_2$	Lactate $S_{cv}O_2$	15% increase in VO_2	AUROC for predicting the VO_2-response: Lactate: 0.68 [0.42 - 0.88, $p = 0.28$) $S_{cv}O_2$: 0.80 [0.54 - 0.95] $p = 0.012$ $P_{cv\text{-}a}CO_2/C_{a\text{-}cv}O_2$: 0.64 [0.37 - 0.85, $p = 0.359$. Responders *versus* non-responders: Lactate: 1.0 ± 0.3 *versus* 1.2 ± 0.2mmol/L $S_{cv}O_2$: 53 ± 7 *versus* 60 ± 7% $P_{cv\text{-}a}CO_2/C_{a\text{-}cv}O_2$: 0.22 [0.17 - 0.22] *versus* 0.23 [0.17 - 0.26]
Shaban et al.[17]	Prospective cohort	50	Shock of any etiology	$P_{cv\text{-}a}CO_2/C_{a\text{-}cv}O_2$	Lactate	28-day mortality	Survivors showed lower $P_{cv\text{-}a}CO_2/C_{a\text{-}cv}O_2$ (0.21 ± 0.19 *versus* 0.42 ± 0.68, $p = 0.013$) and lactate (3.8 ± 1.9 *versus* 7.2 ± 4.4mmol/L, $p < 0.001$) than non-survivors. $P_{cv\text{-}a}CO_2/C_{a\text{-}cv}O_2$ and lactate AUROCs for mortality were 0.728 and 0.811 respectively
Valenzuela Espinoza et al.[25]	Prospective cohort	20	Septic shock	$P_{cv\text{-}a}CO_2/C_{a\text{-}cv}O_2$	Lactate	28-day mortality	Lactate was higher in non-survivors than in survivors (6.8 ± 9.3 *versus* 1.5 ± 0.5mmol/L, $p = 0.03$) but $P_{cv\text{-}a}CO_2/C_{a\text{-}cv}O_2$ was similar (2.7 ± 1 *versus* 2.4 ± 1.1, $p = 0.58$).
Gao et al.[26]	Retrospective cohort	145	Septic shock	$P_{cv\text{-}a}CO_2/C_{a\text{-}cv}O_2$	Lactate clearence ratio (LCR)	28-day mortality	AUROC of $P_{cv\text{-}a}CO_2/C_{a\text{-}cv}O_2$ and lactate clearance rate were not significantly different. AUROC of $P_{cv\text{-}a}CO_2/C_{a\text{-}cv}O_2$ was 0.862 [0.795 - 0.914]). AUROC of combined $P_{cv\text{-}a}CO_2/C_{a\text{-}cv}O_2$ and LCR was greater than either $P_{cv\text{-}a}CO_2/C_{a\text{-}cv}O_2$ or LCR alone (0.919 [0.862 - 0.958]).
He et al.[27]	Prospective cohort	61	Septic shock	$P_{cv\text{-}a}CO_2/C_{a\text{-}cv}O_2$	Lactate	ICU mortality	Similar $P_{cv\text{-}a}CO_2/C_{a\text{-}cv}O_2$ (1.7 ± 1.1 *versus* 2.8 ± 2.1, $p = 0.106$) and lactate (3.5 ± 2.4 *versus* 3.0 ± 1.3, $p = 0.792$) in survivors and non-survivors. Multivariate logistic binary regression for ICU mortality: $P_{cv\text{-}a}CO_2/C_{a\text{-}cv}O_2$: 1.722, 95%CI 1.251 - 2.50, $p = 0.024$ Lactate: -0.266, 95%CI -0.514 - 1.14, $p = 0.193$

Continue...

...continuation

Reference	Study design	N	Type of ICU-population	Variables of interest	Comparator	Main outcome	Main results
Mesquida et al.[28]	Prospective cohort	52	Septic shock	$P_{cv-a}CO_2/C_{a-cv}O_2$	Lactate	ICU mortality	$P_{cv-a}CO_2/C_{a-cv}O_2$ and lactate correlated (r = 0.73, p < 0.001). Lower $P_{cv-a}CO_2/C_{a-cv}O_2$ in survivors (1.4 ± 0.5 *versus* 1.9 ± 0.9, p = 0.039) but no differences in lactate (2.8 ± 1.1 *versus* 7.7 ± 9.2mmol/L, p = 0.8).
Moussa et al.[29]	Prospective cohort	308	Cardiac surgery	$P_{cv-a}CO_2/C_{a-cv}O_2$	Lactate	Major cardiac and noncardiac complications occurring in the 48 hours following surgery	$P_{cv-a}CO_2/C_{a-cv}O_2$ was similar between groups at second hour after ICU admission in (p = 0.229) but lactate was higher (p = 0.014) in patients who developed at least one of these outcomes.
Mukai et al.[30]	Prospective cohort	110	Cardiac surgery	$P_{cv-a}CO_2/C_{a-cv}O_2$	$P_{cv-a}CO_2/C_{a-cv}O_2$	Incidence of postoperative severe adverse events	$P_{cv-a}CO_2/C_{a-cv}O_2$ had higher AUROC than lactate for prediction of postoperative major organ morbidity and mortality after cardiac (0.69 [0.54 -0.83] *versus* 0.61 [0.48 - 0.74].
Ospina-Tascón et al.[31]	Prospective cohort	135	Septic shock	$P_{cv-a}CO_2/C_{a-cv}O_2$	Lactate	28-day mortality	Survivors had lower $P_{cv-a}CO_2/C_{a-cv}O_2$ (1.37 ± 0.20 *versus*. 2.23 ± 0.81) and lactate (2.7 ± 0.6 *versus* 5.4 ± 1.2mmol/L) than non-survivors. Multivariate logistic regression for mortality, $P_{cv-a}CO_2/C_{a-cv}O$: had RR 1.61 [0.92 - 2.82], p = 0.10, and lactate 1.15, [0.96 - 1.38], p = 0.13
Saludes et al.[32]	Prospective cohort	20	Shock of any etiology	$P_{cv-a}CO_2/C_{a-cv}O_2$	Lactate	ICU mortality	Lactate and $P_{cv-a}CO_2/C_{a-cv}O_2$ were higher in non-survivors than in survivors (3.4 [2.1 - 9.0] *versus* 1.7 [1.1 - 3.0] mmol/L, p < 0.001 and 2.23 [1.86 - 2.80] *versus* 1.46 [1.21 - 1.89] p < 0.01)-
Zhou et al.[33]	Retrospective cohort	144	Septic shock	$P_{cv-a}CO_2/C_{a-cv}O_2$	Lactate	28-day mortality	$P_{cv-a}CO_2/C_{a-cv}O_2$ and lactate showed similar AUROC (0.755 and 0.742) Cox multivariate survival analysis showed that $P_{cv-a}CO_2/C_{a-cv}O_2$ and lactate at 6 hours were independent predictors of outcome (RR 2.026, [1.221 - 3.361], p = 0.006, and 2.177 [1.749 - 2.711], p < 0.001) but not at 0-h (0.816, [0.477 - 1.395] p = 0.457, and 0.840 [0.691 - 1.020] p = 0.079)
Fuentes-Gómez et al.[34]	Retrospective cohort	110	Septic shock	$P_{cv-a}CO_2/C_{a-cv}O_2$	Lactate	ICU mortality	Non-survivors had higher lactate (4.5 [1.4 - 7.7] *versus* 1.4 [0.7 - 2.1]mmol/L, p < 0.05) and $P_{cv-a}CO_2/C_{a-cv}O_2$ (1.6 [0.8 - 2.5] *versus* 1.1 [0.7 - 1.6] p < 0.001) than survivors.

N - number of participants; $P_{mv-a}CO_2/C_{a-mv}O_2$ - mixed venous minus arterial carbon dioxide pressure to arterial minus mixed venous oxygen content ratio; $C_{a-mv}O_2$ - arterial minus mixed venous oxygen content ratio; $S_{mv}O_2$ - mixed venous oxygen saturation; $P_{cv-a}CO_2/C_{a-cv}O_2$ - central venous minus arterial carbon dioxide pressure to arterial minus central venous oxygen content ratio; ICU - intensive care unit; RR - relative risk; AUROC - area under the receiver-operating characteristic curve; CI - confidence interval; SD - standard deviation; $S_{cv}O_2$ - central venous oxygen saturation. Values are shown as mean ± standard deviation or median [95%CI].

Appendix 4S - Funnel plots of $P_{cv-a}CO_2/C_{a-cv}O_2$ (Panel A) and arterial lactate (Panel B) in survivors and non-survivors excluding studies in which the ratio was calculated from mixed venous samples.

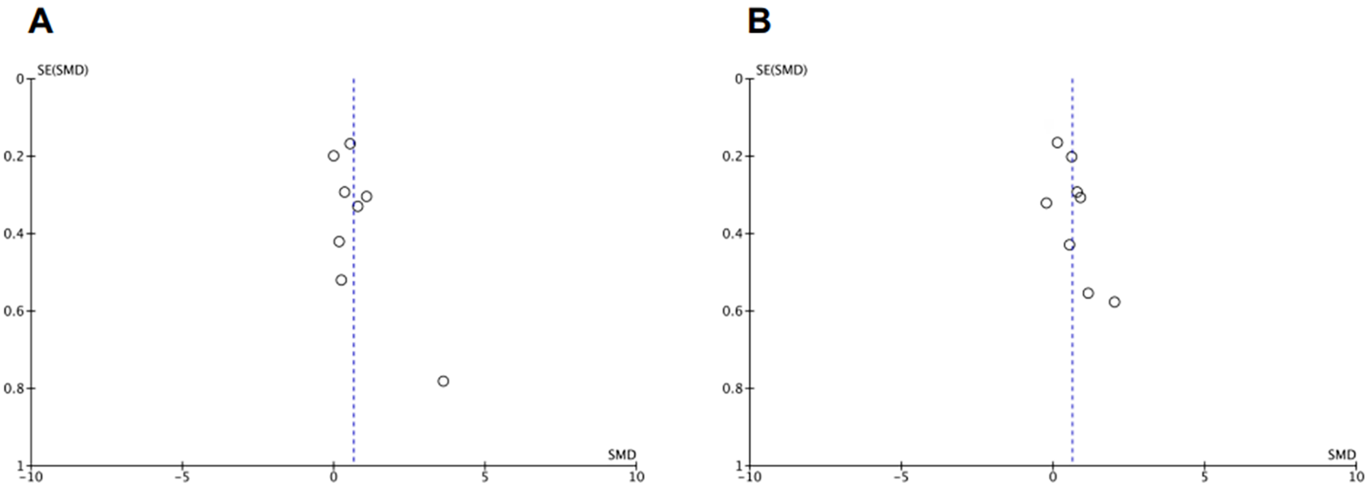

Appendix 5S - Forest plots of Pcv-aCO2/Ca-cvO2 (Panel A) and arterial lactate (Panel B) in survivors and non-survivors excluding studies in which the ratio was calculated from mixed venous samples.

A

	Non Survivors			Survivors				Std. Mean Difference
**Study or Subgroup**	**Mean**	**SD**	**Total**	**Mean**	**SD**	**Total**	**Weight**	**IV, Random, 95% CI**
Dubin 2018	1.3	0.68	13	1.17	0.65	10	11.1%	0.19 [-0.64, 1.01]
Fuentes-Gómez 2018	1.38	2.91	39	1.35	3.25	71	16.3%	0.01 [-0.38, 0.40]
He 2017	2.8	2.1	12	1.7	1.1	49	13.2%	0.81 [0.16, 1.46]
Mesquida 2018	2.2	0.73	21	1.5	0.57	31	13.8%	1.08 [0.48, 1.67]
Saludes 2017	2.28	0.24	9	1.51	0.17	11	5.5%	3.61 [2.08, 5.14]
Shaban 2017	0.42	0.68	31	0.21	0.19	19	14.1%	0.38 [-0.20, 0.95]
Valenzuela 2019	2.7	1	5	2.4	1.1	15	9.1%	0.27 [-0.75, 1.28]
Zhou 2017	3.22	1.22	74	2.61	0.99	70	16.9%	0.54 [0.21, 0.88]
**Total (95% CI)**			**204**			**276**	**100.0%**	**0.65 [0.22, 1.08]**

Heterogeneity: $Tau^2 = 0.25$; $Chi^2 = 27.49$, df = 7 (P = 0.0003); $I^2 = 75\%$
Test for overall effect: Z = 2.96 (P = 0.003)

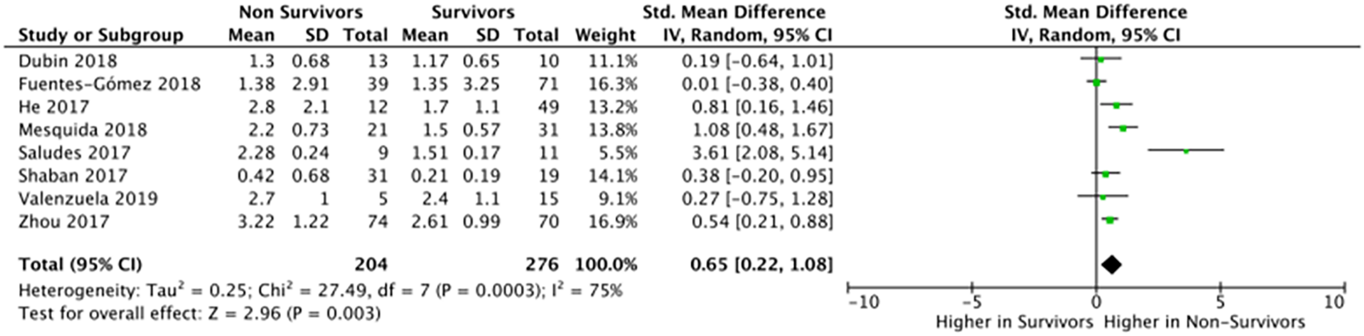

B

	Non-Survivors			Survivors				Std. Mean Difference
**Study or Subgroup**	**Mean**	**SD**	**Total**	**Mean**	**SD**	**Total**	**Weight**	**IV, Random, 95% CI**
Dubin 2018	3.2	2.8	13	1.9	1.2	10	10.1%	0.55 [-0.29, 1.40]
Fuentes-Gómez 2018	4.8	3.6	39	3.06	2.25	71	16.7%	0.62 [0.22, 1.02]
He 2017	3	1.3	12	3.5	2.4	49	13.0%	-0.22 [-0.85, 0.41]
Mesquida 2018	7.5	5.7	21	3.9	3.3	31	13.9%	0.80 [0.23, 1.38]
Saludes 2017	4.48	1.75	9	1.88	0.5	11	7.2%	2.03 [0.91, 3.16]
Shaban 2017	7.2	4.4	31	3.8	1.9	19	13.5%	0.91 [0.31, 1.51]
Valenzuela 2019	6.8	9.3	5	1.5	0.5	15	7.6%	1.15 [0.06, 2.24]
Zhou 2017	5.25	1.71	74	5.01	1.98	70	17.9%	0.13 [-0.20, 0.46]
**Total (95% CI)**			**204**			**276**	**100.0%**	**0.62 [0.25, 1.00]**

Heterogeneity: $Tau^2 = 0.18$; $Chi^2 = 21.56$, df = 7 (P = 0.003); $I^2 = 68\%$
Test for overall effect: Z = 3.25 (P = 0.001)

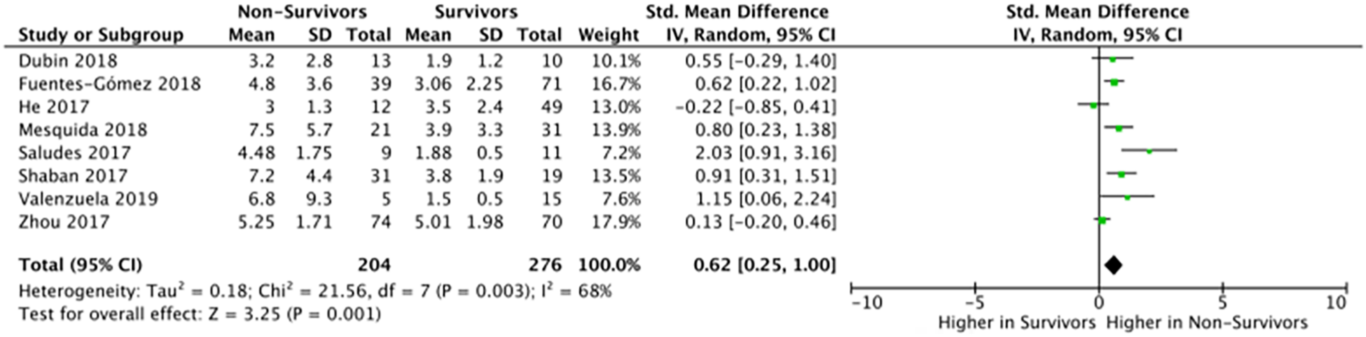

Supplement: Supplementary file 1 [file rbti-34-02-0279-suppl01.pdf]
